# Plg-R_KT_ facilitates plasminogen incorporation and restrains thrombus growth under arterial shear in mice

**DOI:** 10.1186/s10020-026-01485-6

**Published:** 2026-05-13

**Authors:** Claire S. Whyte, Dean Kavanagh, Ausra S. Lionikiene, Steve P. Watson, Robert J. Parmer, Lindsey A. Miles, Nicola J. Mutch

**Affiliations:** 1 Aberdeen Cardiovascular and Diabetes Centre, Institute of Medical Sciences, School of Medicine, Medical Sciences and Nutrition, University of Aberdeen, Aberdeen, AB25 2ZD UK; 2 Department of Cardiovascular Sciences, College of Medicine and Health, University of Birmingham, Birmingham, UK; 3 Department of Medicine (9111H), University of California San Diego, and Veterans Administration San Diego Healthcare System, 3350 La Jolla Village Drive, San Diego, CA 92161 US; 4 Department of Cell and Molecular Biology, Scripps Research, 10550 N. Torrey Pines Rd., SP30-3020, La Jolla, CA 92037 USA

## Abstract

Platelets bind plasminogen facilitating surface-bound plasmin generation. We previously reported that the plasminogen receptor, Plg-R_KT_, retains plasminogen on activated platelets. Here, we investigate the significance of the interaction of Plg-R_KT_ on thrombus formation, growth and stability. Whole blood from Plg-R_KT_^−/−^ or littermate Plg-R_KT_^+/+^ mice was flowed over collagen/tissue factor-coated microfluidic biochips at 250 or 1000 s^−1^ to reflect venous and arterial shear rates. AlexaFluor488-fibrinogen and Dylight633-labelled-plasminogen accumulation was monitored in real-time by fluorescence microscopy in the presence or absence of tissue plasminogen activator (tPA). At 1000 s^−1^, plasminogen accumulation was reduced in thrombi formed from Plg-R_KT_^−/−^ mice compared to Plg-R_KT_^+/+^ mice. Fibrin(ogen) accumulation in Plg-R_KT_^−/−^ mice persisted for the duration of the experiment, indicating impaired fibrinolysis compared to Plg-R_KT_^+/+^ mice. Mice were subjected to FeCl_3_ carotid artery model of thrombosis followed by tPA infusion. Initial platelet deposition was faster in Plg-R_KT_^−/−^ mice compared to Plg-R_KT_^+/+^ mice. Fibrin(ogen) accumulation and persistence was enhanced in Plg-R_KT_^−/−^ mice indicating impaired fibrinolysis. We demonstrate for the first time that under arterial shear, Plg-R_KT_ facilitates plasminogen incorporation and limits both platelet recruitment to the forming thrombus and fibrin accumulation. These data highlight that Plg-R_KT_ and potentially plasmin on the platelet surface regulate arterial thrombus growth.

**Supplementary Information:**

The online version contains supplementary material available at 10.1186/s10020-026-01485-6.

## Key points

Plg-RKT regulates initial platelet deposition and fibrin(ogen) accumulation into thrombi thereby down-regulating thrombolysis.

Plg-RKT functions to restrain thrombus growth and modify the evolving architecture.

**Supplementary Information:**

The online version contains supplementary material available at 10.1186/s10020-026-01485-6.

## Introduction

Colocalization of plasminogen alongside tissue plasminogen activator (tPA) or urokinase (uPA) on fibrin or cellular membranes catalyses conversion to the key fibrinolytic enzyme, plasmin and protects from inhibition by its principal inhibitor, α_2_antiplasmin (Urano et al. 2018). Plasminogen has numerous binding partners on diverse cell types which share the common feature of a C-terminal lysine residue (Miles and Parmer 2013). The role of platelets in localising plasminogen has previously been demonstrated by our laboratories and others on both human (Whyte et al. 2015, 2021a) and murine platelets (Whyte et al. 2021a; Brzoska et al. 2017, 2015). Plg-R_KT_ is the only known transmembrane plasminogen receptor with a readily available C-terminal lysine in its extracellular domain (Andronicos et al. 2010). This receptor supports the conformational change of plasminogen into an open, more readily activated form (Quek et al. 2023) facilitating activation by both tPA (Andronicos et al. 2010) and uPA (Lighvani et al. 2011). Previously, we demonstrated that human and murine platelets expose Plg-R_KT_ upon activation and that this receptor supports approximately 40% of the plasminogen binding to the stimulated platelet surface (Whyte et al. 2021a). The role of Plg-R_KT_ in localising plasminogen within the dynamic thrombus microenvironment has not been established. Here, we utilise global Plg-R_KT_^−/−^ mice (Miles et al. 2017) to define the importance of this receptor in thrombolysis using ex vivo whole blood microfluidic and in vivo FeCl_3_ injury models. These data show for the first time that Plg-R_KT_ modulates initial platelet accumulation into thrombi formed under arterial shear, suggesting that plasminogen localisation via this receptor serves as a check-point mechanism to limit thrombus growth.

## Methods

### Study approval

All animal experiments were carried out in accordance with the U.K. Animals Scientific Procedures Act, 1986 and were approved by University of Aberdeen and University of Birmingham ethics committee and performed under the University of Aberdeen and University of Birmingham UK Home Office Licences, PP9926175 and PP9677279 respectively.

### Plg-R_KT_^−/−^ colony

Mice deficient in Plg-R_KT_ were generated as described (Miles et al. 2017). Plg-R_KT_^+/−^ mice were crossed to obtain Plg-R_KT_^−/−^ mice and Plg-R_KT_^+/+^ littermate controls. All mice were in the C57Bl/6 J background. Genotyping was performed by TransnetYX (Cordova, USA). In total of 92 mice were used to conduct this study.

### Collection of blood

Aged- and sex-matched Plg-R_KT_^−/−^ mice and Plg-R_KT_^+/+^ littermate controls were culled by CO_2_. Blood was collected via the inferior vena cava into 3.2% sodium citrate or acid citrate dextrose (ACD, 80 mM trisodium citrate, 52 mM citric acid and 183 mM glucose).

### Isolation of washed platelets

Whole blood was taken into ACD and was added to modified Tyrode’s buffer (134 mM NaCl, 2.9 mM KCl, 0.34 mM Na_2_HPO_4_.12H_2_O, NaHCO_3,_ 1 mM MgCl_2,_ 5 mM gLucose, 20 mM HEPES, pH 7.3) at a ratio of 1:5. Platelet-rich plasma (PRP) was obtained by centrifuging at 200 × g for 5 min. The remaining erythrocyte layer was diluted with Tyrode’s buffer and centrifuged at 200 × g for 6 min and the resulting PRP fractions combined. A platelet pellet was obtained by centrifuging at 1000 × g for 6 min in the presence of 10 µg/mL prostaglandin I_2._ Platelets were counted on a Sysmex Haematology Analyser and adjusted to 5 × 10^8^ platelets/mL.

### Turbidity assay

PRP (30%) was obtained by centrifuging at 200 × g for 10 min from Plg-R_KT_^−/−^ mice or Plg-R_KT_^+/+^ littermate controls and was clotted by recalcifying with 10.6 mM CaCl_2_ in the presence of tPA (1 nM). Change in absorbance at 405 nm was monitored every min for 8 h at 37 °C on a Varioskan Lux Microplate reader.

### Western blotting

Platelet poor plasma or washed platelets (5 × 10^8^ platelets/mL) were exposed to three freeze/thaw cycles before being separated on 4–12% NuPAGE Bis–Tris gels under non-reducing conditions and then transferred to PVDF membrane, as described (Simpson et al. 2023). Plasminogen was detected using an in-house rabbit polyclonal antibody (1:4000, R177) (McWilliam et al. 1996) (RRID: AB_3740853) and a swine anti-rabbit HRP-conjugated secondary antibody (1:2000, Dako, (Agilent Cat# P0217, RRID:AB_2728719)). Mouse plasminogen (50 ng, Enzyme Research Laboratories) was included as a control. Fibrinogen was detected using a sheep anti-human fibrinogen HRP conjugated antibody (1:4000, Enzyme Research Laboratories, Cat# SAFG-HRP, RRID: AB_3741657). Quantification was performed using ImageJ (NIH).

### Ex vivo whole blood thrombolysis model

Recalcified whole blood thrombi were formed by perfusing for 4 min at 1000 s^−1^ on a collagen (100 ng) and tissue factor (300 pM)-coated surface in Cellix Vena8 Fluoro + biochips with added AlexaFluor 488 (AF488)-labelled human fibrinogen (16.7 µg/mL, (Molecular Probes, F13191)) or DyLight633-labelled human plasminogen (0.9 µM (prepared in-house using Thermo Scientific DyLight666 labelling kit, 53046 and Enzyme Research Laboratories human Glu-plasminogen, HPG2001)) ± tPA (30 nM, Actilyse). This was followed by perfusing tPA (30 nM) in Hepes buffer pH 7.45 (136 mM NaCl, 2.7 mM KCl, 10 mM Hepes, 2 mM MgCl_2_, glucose 0.1%) for up to 26 min to visualise fibrin formation and lysis. In some instances, platelets were post-stained with AlexaFluor 568 Annexin V (1:20 dilution (Molecular Probes, A13202)). For lysis experiments, the initial AF488-fibrinogen was included for the first 4 min post-blood perfusion. Thrombus formation and lysis was imaged every 10 s on an EVOS M5000 microscope using a 60 X oil objective. Images were recorded using consistent imaging settings for the experiment. Thresholding was set based on the initial background signal obtained with values above this being set a positive area using QuPath.

### FeCl_3_ injury model of thrombosis and thrombolysis

Thrombosis was induced in the carotid artery in Adult C57Bl/6 mice (8–10 weeks, mixed sex, 5–6 males and females/group, outliers were excluded as detected using GraphPad Prism 10). All procedures were carried out in accordance with a supporting home office licence PP9677279 and with local ethical approval. Anaesthesia was induced via intraperitoneal administration of ketamine hydrochloride (100 mg/kg) and medetomidine hydrochloride (100 mg/kg) and maintained as required via additional intraarterial administration. Prior to exposure of the carotid, AlexaFluor647-labelled human fibrinogen (100 μg (Molecular Probes, F35200)) and DyLight488-GP1bβ antibody (Emfret Cat# X488, RRID:AB_2890921) were administered to visualise fibrinogen and platelets, respectively. Subsequently, right carotid arteries were surgically exposed and a small strip (5 × 10 mm) of flexible black plastic sheet was placed under the carotid to minimize erroneous signal and to ensure that the imaging was from the required vessel alone. To initiate thrombosis, filter paper (2 × 1 mm) soaked with 30% FeCl_3_ in PBS was applied for three min on the arterial adventitia (Li et al. 2016). The vessel was imaged in a single z-plane for 30 min in real-time using a Zeiss Examiner Z1 upright intravital microscope with an RMS-thread adapted UPlanFLN 4X objective and a spinning disk confocal (Yokogawa CSU-X1, Yokogawa Electric Corporation). After 5 min of imaging thrombus formation, tPA (1 mg/kg body weight) was infused into the jugular vein. Imaging data was captured digitally using Slidebook6 software (Intelligent Imaging Innovations). Thrombus formation was measured as fluorescence signal of DyLight488-GP1bβ for platelets and AlexaFluor647-labelled fibrinogen over the experimental time frame using ImageJ (NIH). Non-carotid regions were removed from the analysis by application of a region of interest, and integrated density was measured for the two relevant channels across a single Z plane. Fluorescence was measured upstream of the treated area, and this value subtracted from the integrated density value to control for background fluorescence. Integrated density was defined as the product of all pixel intensities within the region of interest.

### Data analysis

Statistical analysis was completed using GraphPad Prism (version 10.2.3). Normally distributed data were analysed using a One-way ANOVA followed by a Bonferroni multiple comparison or an unpaired students t-test. Data that was not normally distributed were using a Kruskal–Wallis followed by a post-hoc Dunn’s multiple comparison test or an unpaired Mann–Whitney test. Image analysis was performed to determine area coverage or integrated density using QuPath 0.4.3 and Image J respectively.

## Results

### Plasminogen levels in platelets are not altered by Plg-R_KT_ deficiency

**Fig. 1:**
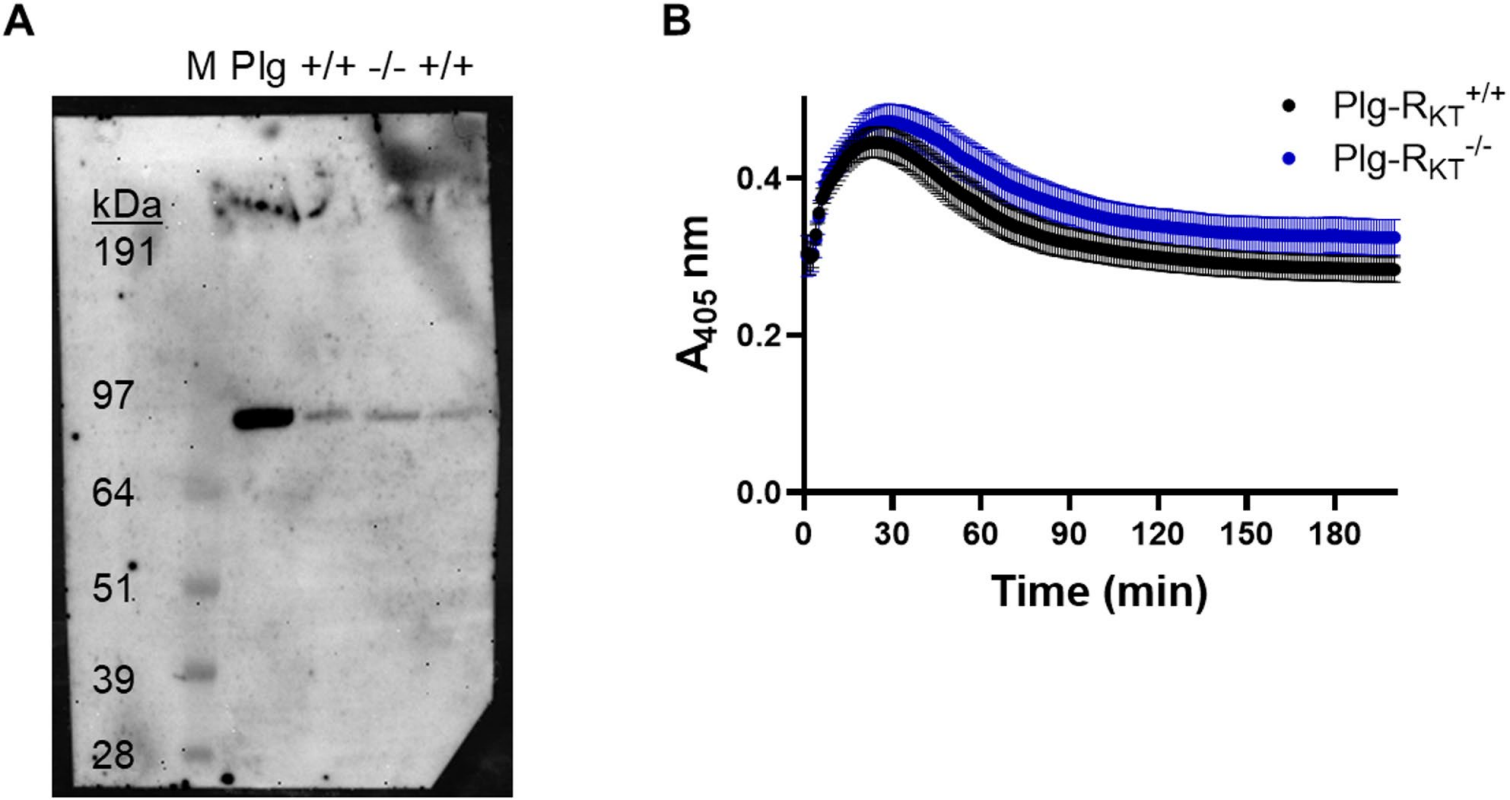
Plg-R_KT_^−/−^ deficiency does not alter platelet plasminogen levels in platelets. **A** Mouse glu- plasminogen (50 ng, Plg), 5 × 10^8^ washed platelets from Plg-R_KT_^+/+^, or Plg-R_KT_^−/−^ mice were separated under non-reducing conditions and detected with a rabbit anti-plasminogen polyclonal antibody, representative blot, *n* = 6. M = molecular weight marker. **B** Platelet-rich plasma (30%) from Plg-R_KT_^−/−^ or Plg-R_KT_^+/+^ mice were clotted by recalcification with 10.6 mM CaCl_2_ in the presence of tPA (1 nM). Change in absorbance at 405 nm was monitored every min for 8 h at 37 °C. Data are mean absorbance over time ± SEM, *n* = 14

Our previous work demonstrated that platelet-derived plasminogen is exposed on activated human and murine platelets (Whyte et al. 2021a), however, the impact of Plg-R_KT_ on the uptake of plasminogen into platelets is unknown. Here, we demonstrate that Plg-R_KT_ does not impact storage of plasminogen within resting mouse platelets (median integrated density [IQR]; Plg-R_KT_^−/−^ 2677 [711.6–4385] vs Plg-R_KT_^+/+^ 1684 [1086–3383] arbitrary units, Fig. 1A). Plg-R_KT_^−/−^ mice demonstrate normal haemostasis, no spontaneous fibrin deposition. In agreement with previous data (Whyte et al. 2021a; Miles et al. 2017), levels of plasminogen (mean integrated density; Plg-R_KT_^+/+^ 13,869 ± 1526 vs Plg-R_KT_^−/−^, 13,424 ± 1681 a.u.) and fibrinogen (Western blotting, mean integrated density; Plg-R_KT_^+/+^ 14,447 ± 2012 vs Plg-R_KT_^−/−^, 13,933 ± 1874 a.u., (*n* = *8*)) in plasma are similar to Plg-R_KT_^+/+^ littermates. Mean platelet counts also did not differ between the groups (Plg-R_KT_^+/+^, 943 ± 78 vs. Plg-R_KT_^−/−^, 834 ± 89 × 10^3^platelets/µL). In line with the similar plasma plasminogen content, lysis of platelet rich plasma clots under static conditions did not differ between Plg-R_KT_^−/−^ mice and Plg-R_KT_^+/+^ controls (median 50% lysis time [IQR]; Plg-R_KT_^−/−^, 76.0 [71–88.8] vs Plg-R_KT_^+/+^, 70.8 [45.4–91.4] min), (Fig. 1B).

### Plasminogen incorporation into thrombi under arterial shear is reduced in the absence of Plg-R_KT_

**Fig. 2:**
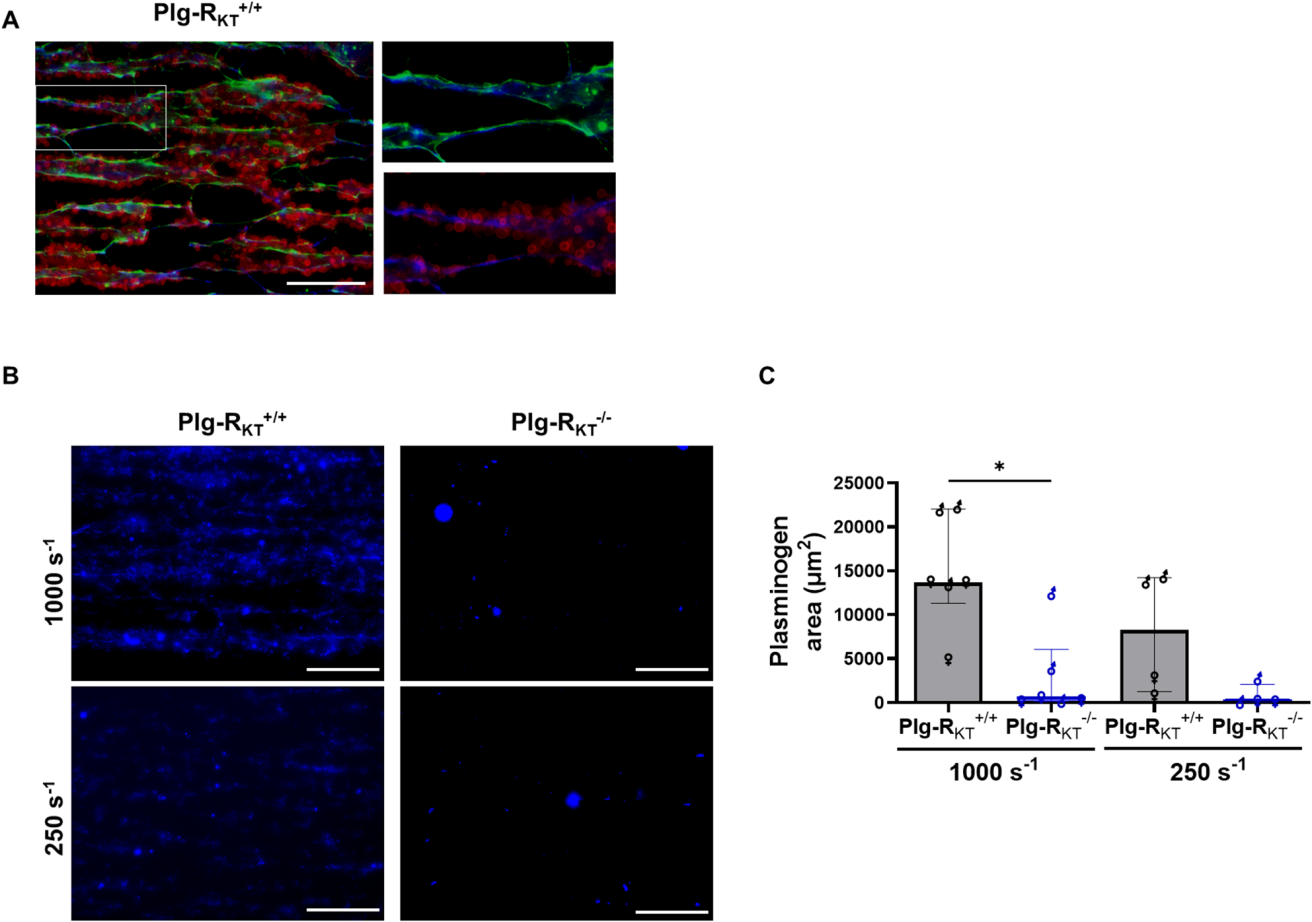
Plasminogen accumulation is significantly reduced in arterial thrombi formed from Plg-R_KT_^−/−^ mice. Whole blood from Plg-R_KT_^+/+^ or Plg-R_KT_^−/−^ mice with AlexaFluor488 fibrinogen (green, GFP Channel) and Dylight633-labelled plasminogen (blue, CY5 Channel) incorporated, was flowed over collagen/tissue factor coated microfluidic chambers at 1000 s^−1^. Thrombi were allowed to form for 4 min before switching to Hepes containing AlexaFluor488 fibrinogen for 4 min. **A** Thrombi formed from blood from Plg-R_KT_^+/+^ mice. Platelets were post-stained with AlexaFluor 568 Annexin V (red, RFP Channel). Images shown were captured at 10 min. Panels on the right are digital magnification showing plasminogen with fibrinogen or platelets. **B** Blood from Plg-R_KT_^−/−^ and Plg-R_KT_^+/+^ mice with added Dylight633-labelled plasminogen was flowed over collagen/tissue factor coated microfluidic chambers at 250 or 1000 s^−1^. Images shown are at the 11 min time point. **C** Quantification of plasminogen positive areas from **B**. ** *P* < 0.01. Data are median ± interquartile range [IQR], *n* = 4–6. Males and females are differentiated by the following symbols; ♂, ♀ respectively

It is possible in the absence of convective removal, the high plasma plasminogen concentration and abundant plasminogen binding sites on fibrin overshadow the influence of Plg-R_KT_ on localising the zymogen. However, i*n vivo*, there is convective removal and extravasation of proteins from the thrombi (Welsh et al. 2016). Therefore, we studied thrombus dynamics in relation to plasminogen accumulation by forming thrombi over tissue factor and collagen coated chips. Whole blood from Plg-R_KT_^+/+^ or Plg-R_KT_^−/−^ mice was perfused over collagen/tissue factor coated microfluidic biochips at low (250 s^−1^) or high (1000 s^−1^) shear. Plasminogen was detected in close proximity to fibrin(ogen) and platelets in thrombi formed from Plg-R_KT_^+/+^ mouse blood (Fig. 2A). In the absence of Plg-R_KT,_ there was a significant reduction in plasminogen incorporated into thrombi at high shear (1000 s^−1^) but not at low shear (250 s^−1^) (Fig. 2B-C). Fibrinogen accumulation did not differ significantly in the absence of tPA (Supplemental Fig. 1).

### The absence of Plg-R_KT_ promotes fibrin(ogen) accumulation and defective fibrinolysis under high shear

**Fig. 3:**
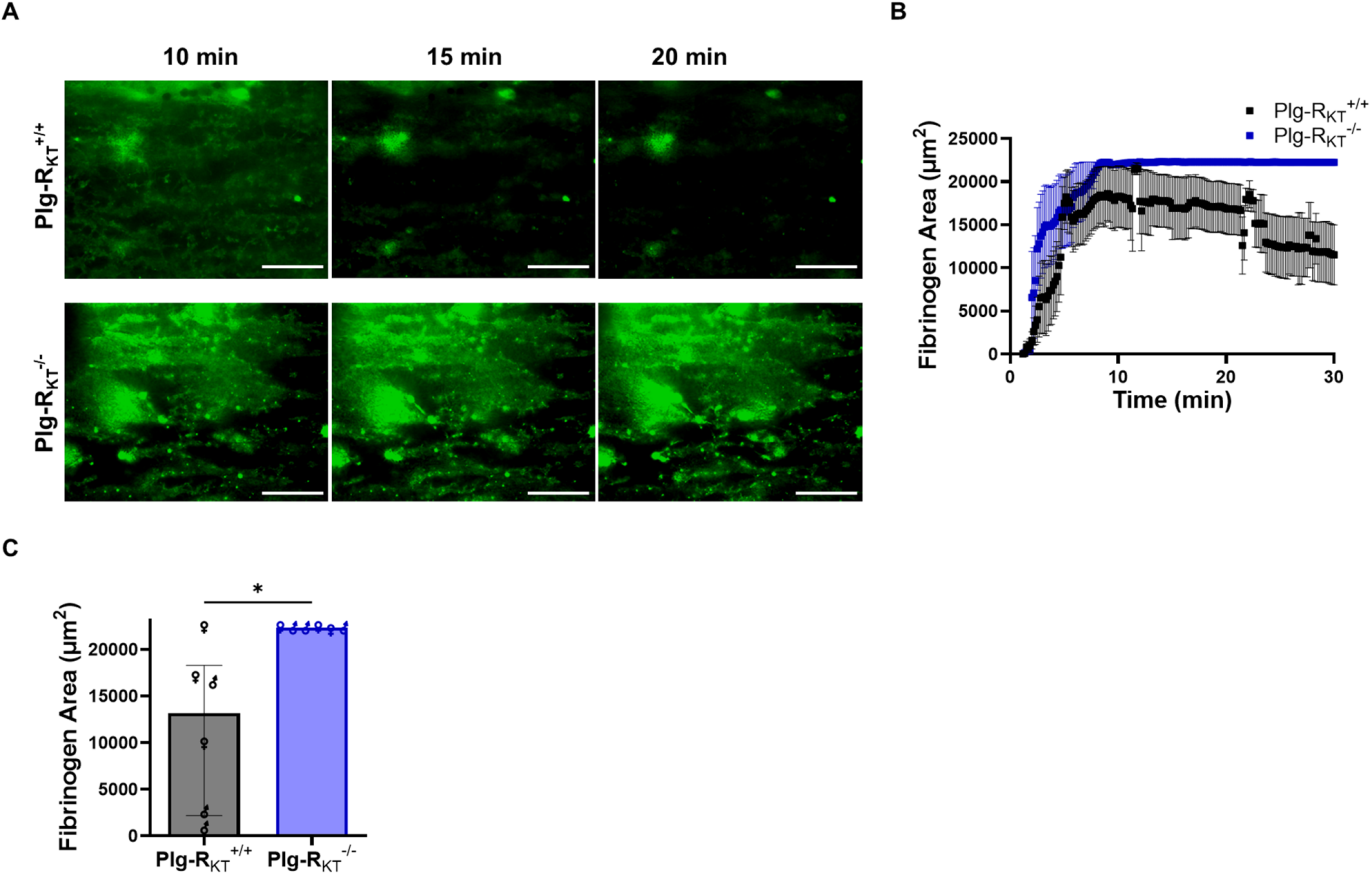
Enhanced fibrin(ogen) accumulation and defective thrombus clearance in the absence of Plg-R_KT_. AlexaFluor488 fibrinogen and tPA (30 nM) was incorporated into whole blood from Plg-R_KT_^−/−^ and Plg-R_KT_^+/+^ mice immediately prior to recalcification and initiation of flow. Thrombi were formed by flowing over collagen/tissue factor coated microfluidic chambers at 1000 s^−1^. Thrombi were allowed to form for 4 min before switching to HEPES buffer containing AlexaFluor488 fibrinogen and 30 nM tPA for 4 min. This was followed by continuous perfusion of tPA for 22 min. **A** representative images **B** quantification of fibrin(ogen) area coverage over time **C** fibrin(ogen) area coverage at 30 min. * *P* < 0.05. Data are median ± [IQR], representative images of *n* = 6, scale bars indicate 40 µm. Males and females are differentiated by the following symbols; ♂, ♀ respectively

To address the impact of reduced plasminogen accumulation on thrombolysis in thrombi formed from Plg-R_KT_^−/−^ mice under high shear, we incorporated a continuous flow of tPA. Fibrin(ogen) area coverage in real-time was quantified to assess the degree of fibrinolysis. At 30 min, the fibrin(ogen) positive area in the field of view was reduced by approximately 20% in thrombi formed from Plg-R_KT_^+/+^ mice under high shear (Fig. 3). In contrast, thrombi formed from the Plg-R_KT_^−/−^ mice demonstrated negligible fibrin(ogen) clearance within the timeframe (Fig. 3). This suggests that the absence of Plg-R_KT_ promotes fibrin(ogen) accumulation and defective fibrinolysis under high shear in mice.

### Initial platelet accumulation is faster in arterial thrombi in the absence of Plg-R_KT_

**Fig. 4:**
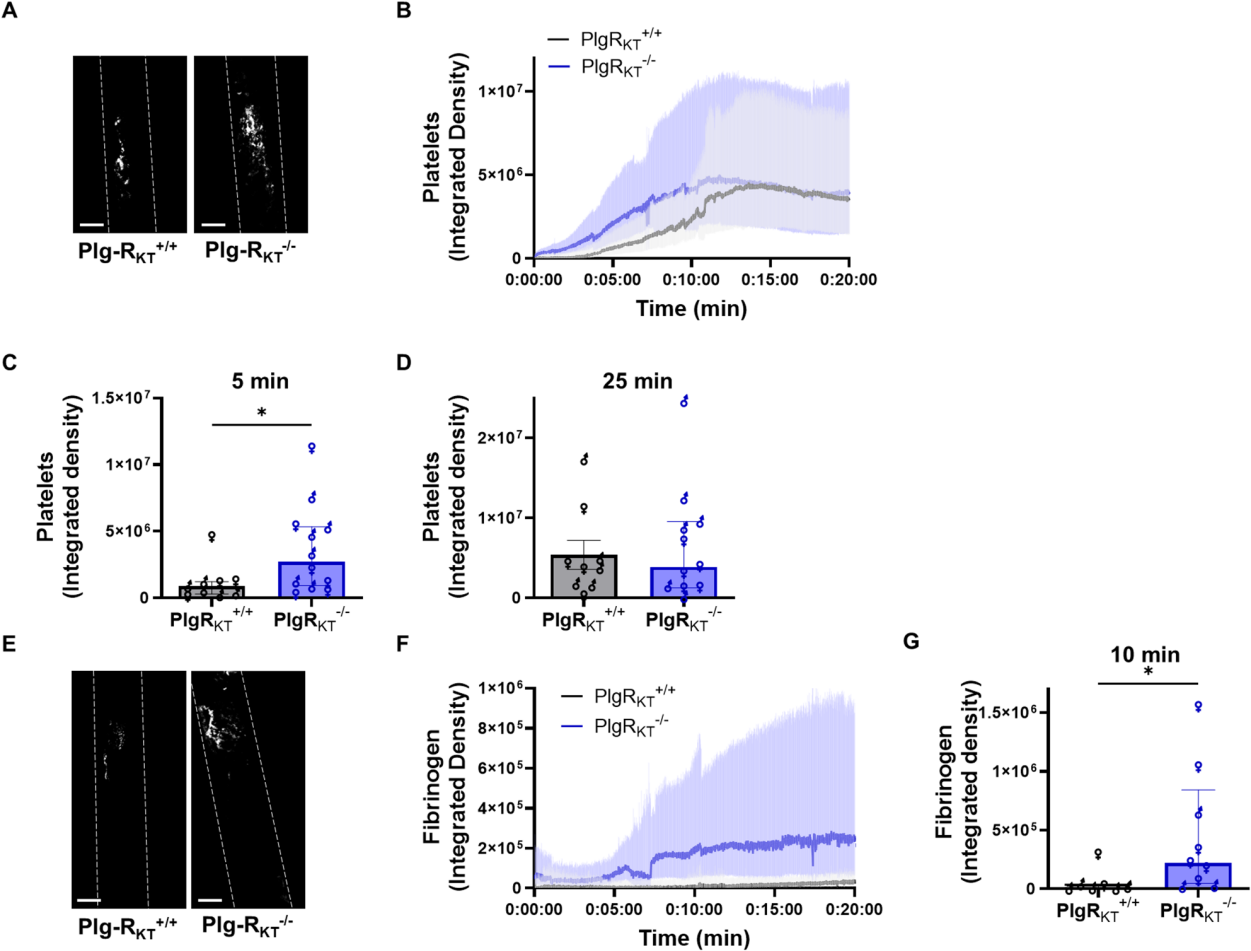
Platelet-rich arterial thrombi form faster in Plg-R_KT_^−/−^ mice compared to Plg-R_KT_^+/+^ mice. Thrombosis was induced in the carotid artery using 30% FeCl_3_ with prior infusion of AlexaFluor647-labelled fibrinogen and DyLight488-GP1bβ antibody to label platelets. After 5 min tPA was infused. Thrombus formation was imaged for 30 min in real-time using a Zeiss Examiner upright intravital microscope equipped with spinning disk confocal. **A** Representative images of platelet accumulation at 5 min. Scale bars indicate 200 µm, dashed lines indicate the vessel wall boundaries. **B** Changes in the fluorescence signal for platelets measured as GP1bβ monitored over time and quantified as the median integrated density ± [IQR] at **C** 5 and **D** 25 min. **E** Representative images of fibrin(ogen) accumulation at 10 min. **F** Changes in fibrinogen integrated density over time, and **G** median integrated density ± [IQR] at 10 min. * *P* < 0.05 determined by Mann–Whitney test, *n* ≥ 7. Males and females are differentiated by the following symbols; ♂, ♀ respectively

To examine the contribution of Plg-R_KT_ to fibrinolysis in vivo, we used a ferric chloride carotid artery injury model of thrombosis and subsequent thrombolysis. Fibrin(ogen) and platelet deposition were monitored over time using AlexaFluor647-labelled fibrinogen and DyLight488-GP1bβ antibody, respectively. After 5 min, a bolus of tPA was injected and thrombus dynamics monitored for a further 25 min. In Plg-R_KT_^−/−^ mice, there was a threefold increase in the initial rate (0—5 min post-injury) of platelet deposition compared to Plg-R_KT_^+/+^ mice (Fig. 4A-C). However, by 25 min, platelet accumulation in Plg-R_KT_^−/−^ mice reached the same level as Plg-R_KT_^+/+^ controls (Fig. 4D).

### Arterial thrombolysis is impaired in Plg-R_KT_^-/-^ mice

Initial rates of fibrin(ogen) accumulation (0—5 min) in arterial thrombi were similar in both the Plg-R_KT_^−/−^ mice and Plg-R_KT_^+/+^ littermate controls (Fig. 4E & F). Following tPA infusion there was an initial decrease in fibrin(ogen) accumulation. However, by 10 min there was a significant increase in fibrin(ogen) accumulation in thrombi formed from Plg-R_KT_^−/−^ mice, approximately 12-fold over that of Plg-R_KT_^+/+^ littermate controls (Fig. 4G). Thrombi formed from the Plg-R_KT_^+/+^ littermates failed to accumulate significant amounts of fibrin(ogen) in the presence of tPA. In contrast fibrin(ogen) persisted for the duration of the experiment (30 min) in the Plg-R_KT_^−/−^ mice. Initial rates of fibrin(ogen) accumulation (0—5 min) in arterial thrombi were similar in the Plg-R_KT_^−/−^ and Plg-R_KT_^+/+^ mice (Fig. 4E & F). Infusion of tPA initially decreased fibrin(ogen) accumulation. However, by 10 min, fibrin(ogen) accumulation increased approximately 12-fold in thrombi formed from Plg-R_KT_^−/−^ mice over that of Plg-R_KT_^+/+^ controls (Fig. 4G), which only accumulated minimal amounts of fibrin(ogen) in the presence of tPA. In contrast, fibrin(ogen) persisted for the duration of the experiment (30 min) in the Plg-R_KT_^−/−^ mice.

## Discussion

The role of platelets in localising plasminogen has previously been demonstrated by our laboratory and others on both washed human (Whyte et al. 2015, 2021a) and murine platelets (Whyte et al. 2021a; Brzoska et al. 2017, 2015). However, i*n vivo* surface binding of proteins is markedly more complex, due to convective removal and extravasation of proteins from the thrombi (Welsh et al. 2016). Under thrombotic challenge with subsequent thrombolysis, we observe that in vivo Plg-R_KT_ regulates fibrin accumulation within the developing thrombus, indicating that local accumulation of fibrinolytic factors is crucial for driving thrombus resolution. We previously observed that C-terminal lysine residues were required for retention of platelet-derived plasminogen released from α-granules upon stimulation (Whyte et al. 2021a). Plg-R_KT_ accounts for approximately 40% of the plasminogen retention on platelets and therefore other receptors with C-terminal lysine residues on the platelet surface and most probably binding to fibrin itself anchored to αIIbβ3 (Whyte et al. 2015, 2021a; Brzoska et al. 2017; Miles and Plow 1985; Ni et al. 2020) could also facilitate plasminogen incorporation. Our data indicate that Plg-R_KT_ exposed on the platelet surface functions to bind exogenous plasma plasminogen or retain platelet-derived plasminogen within the thrombi by tethering it to the activated membrane to overcome convective removal. Therefore, in the absence of this receptor, plasminogen is less efficiently incorporated into thrombi and thus impacts on the fibrinolytic balance and subsequent stability. Our previous data, from human thrombi formed in a Chandler loop demonstrated that tPA and plasminogen predominantly localise to the platelet and leukocyte-rich head rather than the fibrin-rich tail (Whyte et al. 2021b; Robbie et al. 1997). The greater impact seen here of Plg-R_KT_ at high shear may reflect a larger contribution of platelets to thrombi formed under higher shear conditions (Whyte and Mutch 2022).

Thrombotic challenge in vivo at arterial shear, revealed, that mice deficient in Plg-R_KT_ show enhanced platelet accumulation in the initial phase following injury compared to Plg-R_KT_^+/+^ littermates. To the best of our knowledge, this is the first in vivo evidence defining the crucial role of plasminogen in limiting platelet recruitment into the forming thrombus. Cell-associated plasmin proteolysis of fibrin has been suggested to loosen the matrix, impeding attachment of platelets and U937 cells under flow (Lishko et al. 2010). Consistently, a fibrin film or shell has been described at the clot surface which prevents platelet adhesion under static conditions (Alkarithi et al. 2025), which is also visible in thrombi obtained after thromboectomy (Meglio et al. 2019). Our in vivo data suggests in the absence of Plg-R_KT_, reduced platelet-bound plasminogen facilitates platelet adhesion to growing thrombi. By 25 min, platelet accumulation had equilibrated in the WT to the same level as observed in the Plg-R_KT_^−/−^ mice. This coincides with reported peak platelet accumulation in arterial thrombosis models (Ryn et al. 2003; Baumgartner 1973). Therefore, localization of Plg-R_KT_ on activated platelets may function to limit initial recruitment via local plasmin generation. Further studies, with specific activity probes are required to show a direct role for plasmin.

The impact on initial platelet and fibrinogen accumulation in arterial thrombi in Plg-R_KT_^−/−^ mice suggests that local accumulation of fibrinolytic factors could be crucial for driving thrombus resolution. Taken together, our data indicate that Plg-R_KT_ retains plasminogen within thrombi by tethering it to activated platelets to overcome convective removal. These data and our earlier work (Whyte et al. 2015, 2021b; Mutch et al. 2010, 2007) suggest that it is the intra-thrombus balance of fibrinolytic proteins rather than the systemic levels that are crucial in dictating thrombus dynamics.

Plasminogen deficiency in mice and humans is not associated with thrombotic complications, although fibrin depositions in other organs and tissues are apparent (Schuster et al. 2007). In a mouse venous thromboembolism model, plasminogen deficiency did not alter thrombus mass (Sang et al. 2025). This may reflect the varying influences of shear rate in venous and arterial thrombi, as we observed no effect on plasminogen deposition with loss of Plg-R_KT_ under venous shear. Interestingly, the importance of plasmin in regulating fibrin accumulation during haemostasis was recently demonstrated in a saphenous vein laser injury model (Ballard-Kordeliski et al. 2024).

Our data indicate that Plg-R_KT_ on activated platelets promotes accumulation of plasminogen into developing arterial thrombi potentially directing fibrinolysis and limiting fibrin accumulation within thrombi. We show for the first time that Plg-R_KT_ serves to limit initial platelet deposition in thrombi formed under arterial shear in mice, suggesting a critical role of local surface-bound plasmin in regulating platelet recruitment. These data highlight the importance of Plg-R_KT_ to shape, remodel and limit arterial thrombus progression.

## Supplementary Information

Supplementary Material 1.

## Data Availability

For original data please contact Dr Claire Whyte (c.s.whyte@abdn.ac.uk).
