# Supplementary material for: Plg-RKT facilitates plasminogen incorporation and restrains thrombus growth under arterial shear in mice
Source: Mol Med. 2026 May 13;32:102. doi: 10.1186/s10020-026-01485-6 (PMC13339278; doi:10.1186/s10020-026-01485-6)
Supplement: Supplementary file 1 — Supplementary Material 1. [file 10020_2026_1485_MOESM1_ESM.docx]

**Supplemental Material**

**
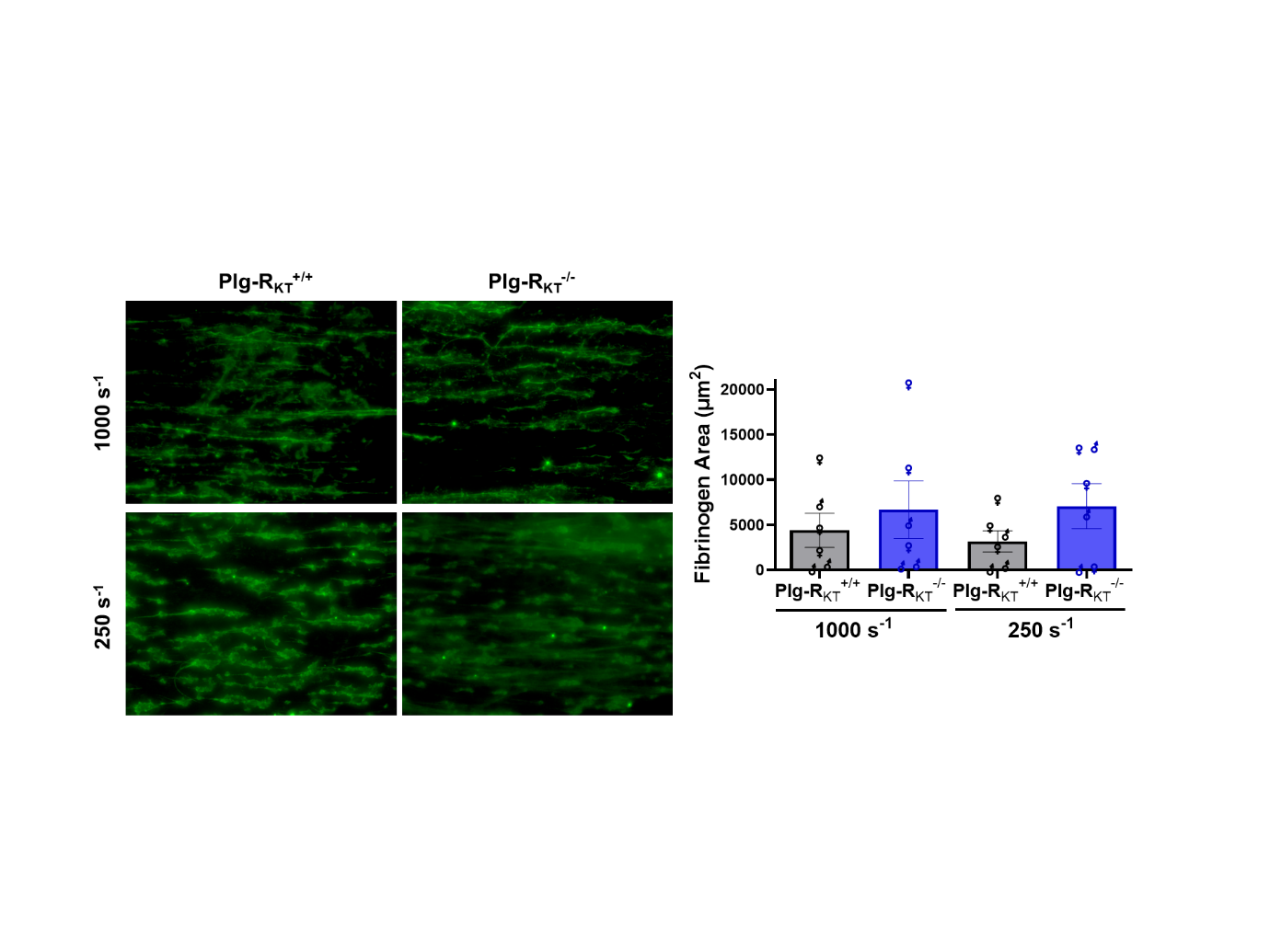
**

**Supplemental Figure 1 -** Whole blood from Plg-R_KT_^+/+^ or Plg-R_KT_^-/-^ mice with AlexaFluor488 fibrinogen and Dylight633-labelled plasminogen incorporated, was flowed over collagen/tissue factor coated microfluidic chambers at 1000 s^-1^. Thrombi were allowed to form for 4 min before switching to Hepes containing AlexaFluor488 fibrinogen for 4 min. (A) Representative images. (B) Fibrinogen positive areas were quantified and data are presented as mean ± SEM, *n* = 6. Males and females are differentiated by the following symbols; ♂, ♀ respectively.
